# Lifestyle changes and glycemic control in type 1 diabetes mellitus: a trial protocol with factorial design approach

**DOI:** 10.1186/s13063-020-4205-7

**Published:** 2020-04-20

**Authors:** Sobiya Sawani, Amna Rehana Siddiqui, Syed Iqbal Azam, Khadija Humayun, Asma Ahmed, Aysha Habib, Sabahat Naz, Mayera Tufail, Romaina Iqbal

**Affiliations:** 1 Department of Community Health Sciences, The Aga Khan University, Stadium Road, PO Box 3500, Karachi, 74800 Pakistan; 2 Department of Pediatrics & Child Health, The Aga Khan University, Stadium Road, PO Box 3500, Karachi, 74800 Pakistan; 3 Department of Medicine, The Aga Khan University, Stadium Road, PO Box 3500, Karachi, 74800 Pakistan; 4 Department of Pathology & Laboratory Medicine, The Aga Khan University, Stadium Road, PO Box 3500, Karachi, 74800 Pakistan

**Keywords:** Type 1 diabetes, Self-management, HbA1c, Log books, Step count

## Abstract

**Background:**

Type 1 diabetes (T1D) has been increasing globally over the past three decades. Self-monitoring of blood glucose is a challenge in both developed as well as developing countries. Self-management guidelines include maintaining logbooks for blood glucose, physical activity, and dietary intake that affect glycated hemoglobin (HbA1c) and a multitude of life-threatening acute complications. Innovative, cost-effective interventions along with beneficial lifestyle modifications can improve home-based self-monitoring of blood glucose in T1D patients. The overall objective of this study is to evaluate the relationship between maintaining log books for blood glucose levels, reinforcement by e-messages, and/or daily step count and changes in HbA1c.

**Methods/design:**

A randomized controlled trial will enroll participants aged 15 years and above in four groups. Each group of 30 participants will be working with a newly designed standard log book for documenting their blood glucose. The first group will be entirely on routine clinical care, the second group will be on routine care and will receive an additional e-device for recording step count (fit bit), the third group will receive routine care and daily motivational e-messages to maintain the log book, and the fourth group along with routine care will receive an e-device for measuring step count (fit bit) and e-messages about maintaining the log book. Patients will be enrolled from pediatric and endocrine clinics of a tertiary care hospital in Karachi. All groups will be followed up for a period of 6 months to evaluate outcomes. Log book data will be obtained every 3 months electronically or during a patient’s clinic visit. HbA1c as a main outcome will be measured at baseline and will be evaluated twice every 3 months. A baseline questionnaire will determine the socio-demographic, nutritional, and physical activity profile of patients. Clinical information for T1D and other co-morbidities for age of onset, duration, complications, hospitalizations, habits for managing T1D, and other lifestyle characteristics will be ascertained. Behavioral modifications for maintaining daily log books as a routine, following e-messages alone, fit bit alone, or e-messages plus using fit bit will be assessed for changes in HbA1c using a generalized estimated equation.

**Discussion:**

The proposed interventions will help identify whether maintaining log books for blood glucose, motivational e-messages, and/or daily step count will reduce HbA1c levels.

**Trial registration:**

ClinicalTrials.gov, NCT03864991. March 6, 2019.

## Background

Type 1diabetes (T1D) is an autoimmune disease in which insulin-producing beta cells in the pancreas are destroyed by the human body pathologically, preventing the body from being able to produce enough insulin to adequately regulate blood glucose levels. The diagnosis is based on clinical factors (age, lack of obesity, and insulin resistance), low C-peptide levels, and insulin antibodies. Substantial destruction of insulin-producing beta cells in the pancreas of T1D patients means that rigorous blood glucose monitoring for adjustment of insulin dosage is required on a daily basis [1].

T1D occurs mostly in the pediatric and adolescent age groups with patients presenting with a hyperglycemia and/or diabetic ketoacidosis episode. Such episodes are also the most common life-threatening adverse effects following diagnosis resulting from suboptimal treatment if insulin dosage is not adjusted and blood glucose is not monitored on a daily basis. The incidence of T1D has been increasing over the past three decades [2]. Management of T1D is a big challenge in resource-constrained low to middle income countries due to the need for daily self-monitoring of blood glucose (SMBG) levels and timely entry of these in a log book [3, 4]. SMBG is an invaluable method for monitoring glycemic status; current guidelines recommend its use in all patients with T1D, type 2 diabetes mellitus (T2DM), or any other forms of diabetes (e.g., gestational diabetes) that require administering multiple subcutaneous insulin injections [5]. A study of children and adolescents with T1D showed that an increased daily frequency of SMBG was significantly associated with lower HbA1c levels (0.2% per additional test per day) along with the added benefit of fewer acute complications such as ketoacidosis [6]. Use of innovative mobile phone applications and regular SMBG have been shown to increase compliance with proposed treatment regimens [7].

Taking an extra 1000 steps per day can help to reduce the risk of cardiovascular events significantly in T1D patients [8]. A wireless wearable e-device accompanied by an application (called the fit bit app) tracks step count by recording data in a mobile phone application [9–11]. Studies conducted in Pakistan report non-adherence of T1D patients to dietary advice (58.5%), physical activity recommendations (42.3%), and prescribed insulin regimens (88.1%), with staggering rates of complications such as diabetic acidosis due to suboptimal treatment [12, 13]. HbA1c levels acts as an indicator of glycemic control and correlate with the occurrence of complications [14]. However, HbA1c levels do not indicate the daily variability of blood glucose. The risk related to daily fluctuation of blood glucose levels can be assessed by a devised method [15, 16]. This study proposes to study maintenance of log books for blood glucose levels and whether motivational e-messages and/or using an e-device for measuring step count, alone or together, can potentially help reduce HbA1c levels in T1D patients. In addition, it will explore any effect of increasing step counts on daily blood glucose levels and reduction in acute complications by maintenance of log books for blood glucose.

### Rationale

Adequate glycemic control along with a healthy lifestyle are associated with improved health outcomes and lower morbidity and premature mortality in diabetes patients. Therapeutic insulin regimens need to be strongly regulated during the adolescent period to prevent complications in T1D patients [17]. Adolescents with poor glycemic control had greater proportions of diabetic retinopathy and micro- and macro-albuminuria than patients who maintained their HbA1c levels in a strict therapeutic range [17]. Adherence to management protocols and insulin regimens had an inverse association with occurrence of acute complications in several studies on T1D patients [18, 19]. Poor compliance with or no insulin regimen were directly associated with severity of diabetic ketoacidosis in T1D patients in Pakistan, who needed longer hospital stays [20]. Family-centered care empowering family members to maintain a blood glucose level record sheet, insulin dosage recording, physical activity, and a meal plan resulted in significant differences in behavior and supervision of adolescents with T1D [21]. At the regional level management of T1D is a big challenge as physicians tend not to adhere to the international treatment guidelines regularly [4, 9]. Several e-devices are available to monitor physical activities and these have been studied globally for use by diabetic patients [6, 9–11]. Most of the studies using and e-device for improving patient outcomes have been performed on T2D patients due to its high prevalence [22–27]. T1D patients are usually in the pediatric age bracket and have increased chances of severe acute complications; therefore, multifaceted approaches involving patients, parents, families, doctors, and stakeholders are required for optimal T1D management [12, 13, 17, 20, 28]. A study done in India assessed the reliability of SMBG logs compared with glucometer memory in children with T1D and the impact on glycemic indicators. Children who maintained accurate records (aged 11.54 ± 4.05 years) had decreased HbA1c levels (from 10.79 ± 2.61% to 7.99 ± 0.84%) and those participants who kept inaccurate records had decreased HbA1c levels (from 10.74 ± 2.60% to 9.01 ± 2.33% over a period of 4 months (*p* < 0.002) [6]. There is a dearth of literature on testing of basic, cost-effective interventions to improve home-based and self-monitoring of blood glucose levels and management of insulin regimens with added beneficial lifestyle modifications. Therefore, the proposed study will record adherence to standard protocols for maintenance of SMBG records by patients, checking HbA1c level every three months, and maintaining an insulin dosage record on a daily basis to determine if these lead to reduction in acute complications in T1D patients, thereby reducing their illness-associated mortality and morbidity.

#### Study purpose

The purpose of this formative research using a factorial design is to evaluate the relationship between maintaining log books for daily blood glucose, reinforcement by e-messages, and/or daily step count using a fit bit device and changes in HbA1c in T1D patients visiting Aga Khan University (AKU) endocrine clinics. In addition, episodes of acute complications will be compared in T1D patients in each intervention arm over a period of 6 months.

#### Study objectives

Primary objectives:

1. To determine the effect of daily mobile phone messages for maintenance of log books and self-monitoring of blood glucose levels on HbA1c levels in T1D patients in comparison to routine clinical care
2. To determine the relationship of wearing an e-device for daily step count with blood glucose levels and changes in HbA1c in T1D patients in comparison to routine clinical care

Secondary objectives:

1. To compare the acute complications of T1D in all groups over a period of 6 months
2. To compare the variability of daily blood glucose measurements using an average daily risk range method

## Methods/design

### Study design

**Fig. 1:**
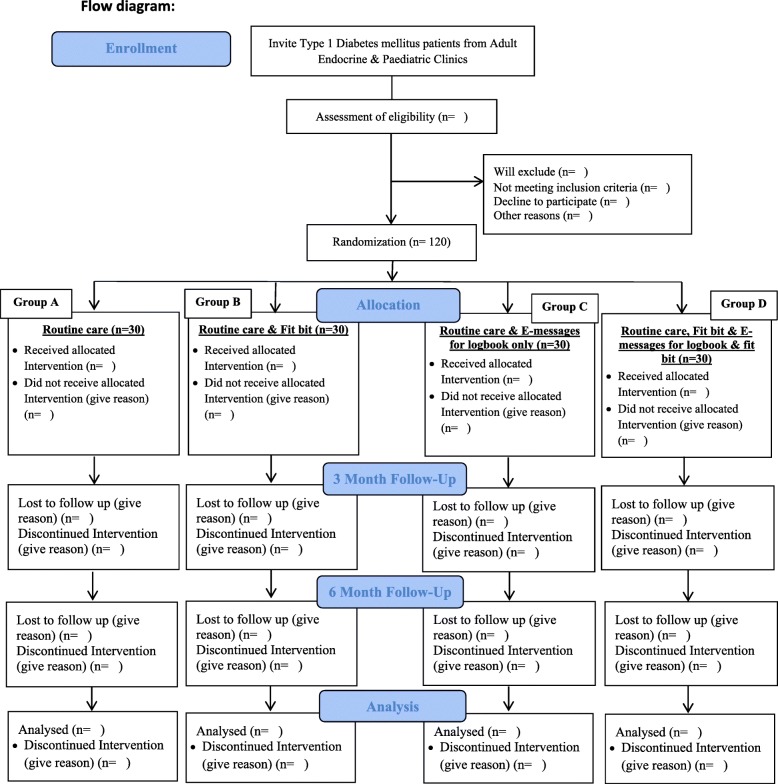
Flow diagram

A factorial design will help to study lifestyle changes for self-management of T1D (Fig. 1). A randomized controlled method will enroll patients in a predefined sequence, assigning them in four groups. All groups will be working with a newly designed standard log book for documenting records for blood sugar and routine insulin dosage and care as provided by doctors, nurses, and nutritionists. The first group will all receive routine care follow-up, the second group will receive an additional e-device for measuring step count (fit bit), the third group will receive routine care and e-messages as reminders to maintain a daily log book, and the fourth group will receive an e-device for measuring step count (fit bit) and e-messages about maintaining the log book, blood glucose levels, and step count in addition to routine care. All groups will be followed up overall for a period of 6 months. HbA1c as a main outcome will be measured at least three times, at baseline, and then once every 3 months.

### Study setting

The study will be conducted in pediatric and adult endocrinology clinics at Aga Khan University (AKU) Karachi, where records of patients are maintained electronically using a heath information management system (HIMS) as well as individual medical record files. Patients under the care of consultant endocrinologists who fulfill the inclusion criteria will be enrolled.

### Study participants

We plan to include all T1D patients aged more than 14 years visiting pediatric and endocrine clinics of AKU. We plan to enroll patients who have been diagnosed with T1D at least 6 months prior to the enrollment.

### Enrolment procedure

Informed consent will be obtained from patients aged 18 or more years and assent from patients aged less than 18 years with additional consent from parents.

### Inclusion and exclusion criteria

Males and females diagnosed with T1D and aged more than 14 years who present to pediatric and endocrine clinics of AKU and have a smart phone or a family member with a smart phone, such as parents or elder sibling, are eligible to participate in the study. However, patients using an “insulin pump” will be excluded. Patients with complicated health conditions such as neurodevelopmental delay and thalassemia will be excluded. Furthermore, patients already using any kind of authentic e-device for measuring step count and/or physical activity will also be excluded from the study.

### Randomization

Unique allocation numbers will be designated by the Clinical Trail Unit (CTU) with computer-generated block randomization, using a block size of four. Sealed opaque identical envelopes will be received by the principal investigator (PI) from the CTU in an order that will be followed to enroll study participants in all four groups. The data collector will be instructed to assign allocated numbers to participants in sequential order as they are enrolled. Upon opening the envelope, the group, A, B, C, and D, will be revealed to the data collector and items related to the appropriate group will be explained to the study participant. Group A will be told to follow routine care, group B will receive an additional e-device for measuring step count (fit bit) and the data collector will download the associated application onto the participant’s cell phone, group C will receive routine care and e-message reminders from the data collector to maintain a daily log book, and group D will receive routine care as well as an e-device for measuring step count (fit bit) together with the associated application (downloaded onto the participant’s cell phone by the data collector) and e-messages from the data collector as reminders to maintain their log book, blood glucose levels, and step count.

### Sample size and sampling technique

Overall, 120 patients with T1D will be randomized into four equal groups for evaluating changes in HbA1C using multiple measurements at multiple time points. Thirty patients in each group will be required in order to achieve 80% power for a two-sided level of significance of 5%, using a standard deviation of change in HbA1c between 1.1% and 2.3% and absolute difference of at least 2% expected over a period of 6 months within a group from baseline as well as when compared across the four groups [29]. A consecutive sampling technique will be used to enroll participants visiting AKU pediatric and endocrinology clinics and fulfilling the eligibility criteria.

### Interventions

**Fig. 2:**
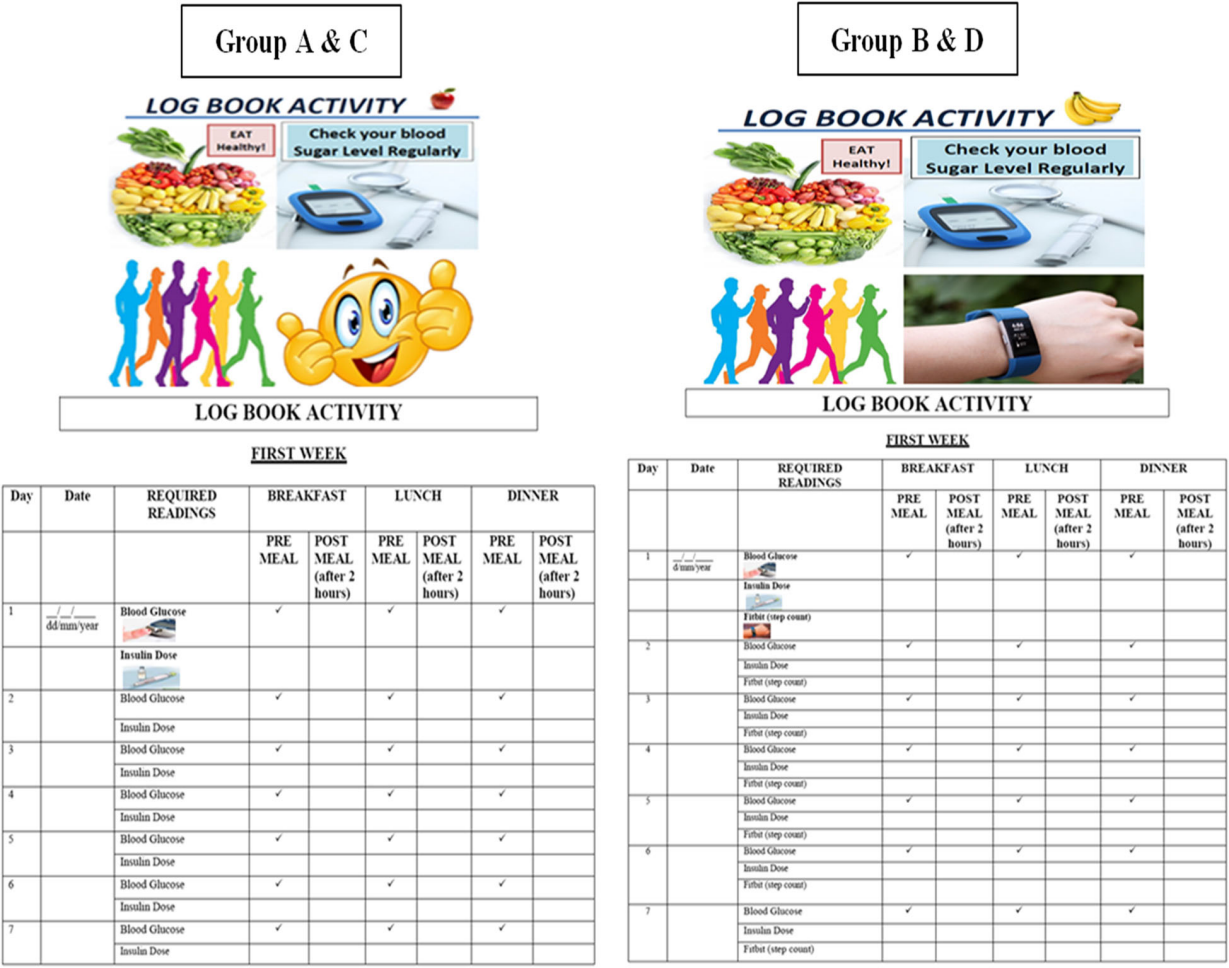
Logbooks for groups A and C to record their daily blood sugar readings and insulin doses and for groups B and D to record daily blood sugar readings, insulin doses, and step count

A predesigned log book file will be provided to all patients in all groups to keep their records of daily blood sugar measurement and insulin therapy (Fig. 2). Random allocation of patients to one of the four groups will be done through support from the CTU at AKU using sealed envelopes. The first intervention group will receive an e-device for daily step count (fit bit). The other two intervention groups will receive reminder mobile e-messages: one will receive only mobile messages (without fit bit) about maintaining the log book for blood glucose and the other will receive the mobile messages for maintaining the log book for blood glucose and for fit bit along with an e-device for measuring step count (Table 1). Fit bit has been shown to have high validity and reliability [10], with the potential advantage over other similar e-devices (accelerometers, pedometers, or actigraphs) of increasing the sustainability of this intervention given that it is cost-effective [27].

**Table 1:** Mobile messages by group

E-MESSEGES FOR GROUP C	E-MESSEGES FOR GROUP D
Remember to check your blood sugars before every meal and record it in your logbook. Keep up the good work.	How many steps have you taken today? Make sure your fitbit is charged to record all that hard work! Good luck and have a healthy day
Don’t forget to check and record your blood sugar and insulin dose in your logbook. This record will help you to discuss it with your doctor and make your sugar levels near to the targets. Have a good day!	Recording your step count can help you to manage your blood sugar, so Fitbit is here to help you to count your steps daily. Don’t forget to charge it. Have a good day!
Keeping an eye on your blood sugar. Knowing this can help you and your doctor to adjust your food choices and insulin doses accordingly. Thanks for checking and recording it in your log book!	Remember! Any type of physical activity you do will help you lower your blood glucose! It’s time to go for a walk and increase your daily steps. Fitbit is here to count your every step so don’t forget to note your steps daily in your logbook. Thank you for all your hard work!
Self-testing of your blood sugar is an important tool in managing your treatment plan and preventing diabetes complications, so try to keep your blood sugar levels record in your logbook as suggested by your physician and help yourself and your doctor to manage your treatment accordingly.	Fitbit is with you to count your steps during housework, outdoor work, job activities, recreational activities, and exercise. So charge you fitbit as required and don’t forget to record your steps daily in logbook. Thanks
Self-monitoring of blood sugar levels provides useful information for diabetes management as it can help you reach your overall treatment goals. So keep checking and recording it in your logbook. Good day!	Improved fitness lowers your blood glucose level, relieves stress, helps insulin work better and strengthens the heart, muscles, and bones. It’s time to set your goals and make your life healthier. Wear your fitbit device all the day and record your steps in log book. Don’t forget to charge it. Stay Healthy!
It is time to check your blood sugar levels and don’t forget to keep recording of your sugar levels and insulin dose in you logbook. Thanks for your all efforts.	If you sit for long hours at work or at home, try to get up once every hour and take a quick walk around the room. Walking is a great way to get fit! So use your fitbit and record all the steps you have taken in the log book. Thanks you for your Hard work!

### Follow-up and other procedures

An orientation meeting of endocrinology unit clinicians and their staff will be conducted prior to initiation of the study to obtain their cooperation for sharing their clinics’ schedules a day prior. HbA1c levels will be laboratory measured at least three times, at baseline and then once every 3 months. Every 3 months, 24-hour dietary recall and physical activity assessments will be performed. Patients’ routine clinic visits are at 3 months and follow up visits will be coordinated accordingly for mid and final data collection and 3-monthly HbA1c testing. Instructions for the e-device (fit bit) will be provided to patients in the respective groups, and its use will be demonstrated to them. A written instruction sheet will be placed in the log book for maintaining the blood glucose and insulin records; also, separate instructions will be given to the e-device (fit bit) groups about charging the device daily at night and wearing it during the day. Study participants/parents will be sent e-messages as reminders for maintaining log books and/or step count. Mobile messages will be sent by trained staff on a daily basis to the assigned groups of patients. Log book data will be obtained every 3 months during doctor visits or through e-messages.

### Outcome measures

The major outcome variable is HbA1c, measured at baseline and 3 and 6 months. HbA1C will be compared with the control (routine) group and with the other groups in this factorial design. This will also be compared within one group: baseline to 3 months and baseline to 6 months. We will explore between-group variation and within-group variation and any interactions between the groups over time.

In addition, secondary outcome variables will be number of all hypoglycemic episodes (blood glucose < 70 mg/dL (3.9 mmol/L) and ketoacidosis episodes (hyperglycemia blood glucose > 11 mmol/L, venous pH < 7.3, or serum bicarbonate < 15 mmol/L, ketonemia blood ß-hydroxybuyrate ≥ 3 mmol/L), taken as a composite variable at baseline and 3 and 6 months [30–32]. Total number of abnormal episodes counted over the follow-up period will be considered as a composite outcome.

### Data collection tools

At baseline we will determine the socio-demographic profile of patients, along with user satisfaction of maintaining log books and using the e-device for measuring step count. Additionally, clinical information for T1D and other co-morbidities, including age of onset, duration, complications, hospitalizations, habits for managing T1D, and other lifestyle characteristics, will be ascertained.

Two other tools will supplement data collection at baseline (0 months) and 3- and 6-month visits; namely, a 24-hour dietary recall [33] and physical activity assessment using both the International Physical Activity Questionnaire (IPAQ) and Youth Physical Activity Questionnaire (YPAQ) [34] (Table 2). The designed questionnaires and tools will be modified for clarity and cultural relevance after pilot testing on at least five T1D patients prior to initiation of the study. All groups will be followed up overall for a total period of 6 months. During these 6 months data for any additional clinic/hospital visit will be obtained from the medical record files of participants.

**Table 2:** Assessments that will be carried out in the study and data collection time points (baseline, 3 months, and 6 months)

Variable	Component	Measurement tools/questions	Baseline	3 month	6 month
Socio-demographic measures		Age, Sex, Weight, Height, Marital Status, Education, Occupation and Parents Education, Occupation and Family history of diabetes	✓		
Health Related Information		History of Diabetes and History if comorbidities	✓		
		Episodes of complications, any hospital admission, Frequency of self-monitoring of blood glucose, frequency of maintaining log book	✓	✓	✓
Behavioral Measure		Frequency of visit to diabetic clinic, frequency of visit to dietitian	✓	✓	✓
	Daily Physical activity of 1 week	Questionnaire adapted from IPAQ and YPAQ	✓	✓	✓
	Barriers to physical activity	Questionnaire adapted from IPAQ and YPAQ	✓	✓	✓
	Dietary Intake	Self-Reported 24- Hour Dietary Recall Questionnaire	✓	✓	✓
Biochemical measures	Pathology	HbA1C	✓	✓	✓

### Ethical considerations

Study participants will be asked to provide informed, written assent/consent prior to participation in this study. Appropriate assent/consent will be obtained by trained data collectors during face to face interviews; the data collectors will also make contact via phone call. Confidentiality of participants will be maintained throughout the follow-up. Study participants will be interviewed in areas separate to the clinical examination room and identification codes will be used to manage the data. This study will not impose any risk or discomfort on any patient or their parents, except for requiring their valuable time; however, this study intends to introduce beneficial lifestyle changes for improving the management of T1D by young adolescents and adults. Ethics approval from Aga Khan University Ethical Review Committee (AKU-ERC) was provided prior to initiating this study.

### Data management

Data collection will be done using digital forms. For any missing or inappropriate information the participant will be contacted on their cell phone number for correction and verification. Every effort will be made to complete the information. All study data will be stored on a protected central server at AKU. Only assigned members of the internal study team can access the respective files.

### Data analysis

We will use an intention-to-treat (ITT) analysis to evaluate changes in HbA1C and abnormal episodes of hypoglycemia and ketoacidosis. Generalized estimating equations will be used, with HbA1C readings at different points in time as our outcome variable. The mean change in HbA1C due to interventions will be estimated at 3 and 6 months, controlling for socio-demographic, clinical, nutritional, and physical activity variables. Secondary outcome variables are reported episodes of acute complications like hypoglycemia and diabetic ketoacidosis and will be recorded from interview-based data conducted at baseline and 3 and 6 months. In addition, these episodes will be further counted for the follow-up period using the daily log book of each participant.

Negative binomial regression will be used to observe the relationship among the four groups and number of episodes, adjusting for any socio-demographic, clinical, nutrition, and physical activity-related variables. If there is an excess number of ‘zeros’—meaning no abnormal episodes—then zero inflated negative binomial regression will be used instead of ordinary negative binomial regression. Twenty-four-hour dietary recall will be converted into total number of calories and percentage carbohydrate intake. Nutrition will be explored for total calories and food groups using factor analysis. Physical activity will be measured using step count data logged on a daily basis. These step counts will be used as time-dependent covariates for the final model. Step counts logged on a daily basis in two groups over a period of 6 months will be obtained from log books. The distribution of these data will be checked and, if needed, will be transformed. Changes in dietary habits and in step count over time will be used as a covariate in the final model. Physical activities reported during the three interviews will be converted into hours per week and categorized by type of activity as mild, moderate, and severe exercise.

In addition, we will explore blood glucose variability on a daily basis to calculate the average daily risk range for hypoglycemia using the standard method developed by Kovatchev and modified for relevant use by other investigators [15, 16].

## Discussion

This randomized controlled trial tests the hypothesis that log book maintenance for SMBG will substantially increase among individuals receiving e-message reminders and will provide evidence for significant correlation between daily step count and blood glucose levels and overall changes in HbA1c in T1D patients. We further expect an interactive effect of e-message reminders and daily step count on blood glucose levels. Such interventions are necessary to bring about positive life style changes among T1D patients. Non-pharmacological interventions like these are user friendly, engaging patients with their home-based care and integrating them with health-care services. A multipronged approach is required to manage diabetes and reduce the severity of complications and our simple interventions include food diaries and physical activity logs together with managing blood sugar by insulin therapy [12, 13, 17, 20, 28].

There is reported evidence of a positive association of e-device with lifestyle changes and diabetes. Most lifestyle intervention studies using an e-device have been focused mainly on education related to advice on diet and physical activity for T2DM, but very few studies have focused on T1D and our study may add further evidence with regard to it [22–27]. Dissemination of this study will provide a potential tool in the form of a log book to be used by practicing physicians in low-resource settings. Additionally, the evidence generated from this study will be shared at various fora to help physicians or providers choose the most suitable lifestyle program for their T1D patients.

### Trial status

This is protocol version 1.0 (June 1, 2018). Recruitment began on October 29, 2018 and recruitment will be completed on approximately March 31, 2020.

## Supplementary information

**Additional file 1.** SPIRIT 2013 checklist: Recommended items to address in a clinical trial protocol and related documents.

## Data Availability

Materials described in this paper pertain to the study protocol only and no raw data are reported. The datasets will be collected and analyzed and can be made available from the corresponding author on reasonable request.

## Abbreviations

AKU: Aga Khan University
CTU: Clinical Trial Unit
HbA1c: Glycated hemoglobin
SMBG: Self-monitoring of blood glucose
T1D: Type 1 diabetes
T2DM: Type 2 diabetes mellitus
